# Mapping Reaction-Diffusion Networks at the Plant Wound Site With Pathogens

**DOI:** 10.3389/fpls.2020.01074

**Published:** 2020-07-16

**Authors:** Stephanie Liu, Yi-Han Lin, Aidan Murphy, Josh Anderson, Nicole Walker, David G. Lynn, Andrew N. Binns, B. Daniel Pierce

**Affiliations:** ^1^ Departments of Chemistry and Biology, Emory University, Atlanta, GA, United States; ^2^ Infectious Diseases and Genomic Medicine Group, J Craig Venter Institute, Rockville, MD, United States; ^3^ Department of Biology, University of Richmond, Richmond, VA, United States; ^4^ Department of Biology, University of Pennsylvania, Philadelphia, PA, United States

**Keywords:** rhizosphere, *Agrobacterium tumefaciens*, confocal microscopy, spatiotemporal mapping, fluorescent biosensor, chemical network

## Abstract

The rich collection of microbes colonizing the plant root making up the rhizosphere function as a multigenomic organ for nutrient distribution. The extent to which its dynamic mutualistic cellular order depends on morphogenic signaling, while likely, remains unknown. We have shown that reaction-diffusion chemical networks constructed with model plant and bacterial metabolites can mimic processes ranging from oxidative burst kinetics to traveling waves and extracellular stationary state reaction-diffusion networks for spatiotemporal ordering of the rhizosphere. Plant parasites and pathogens can be limited by host attachment require dynamic informational networks and continue to provide insight into what controls the rhizosphere. Here we take advantage of *Agrobacterium tumefaciens*, a plant pathogen with a gated receptor that requires simultaneous perception of two plant metabolites. Genetic manipulations have created receptors allowing each metabolite concentration to be correlated with pathogen behavior. The development of the florescent strains used here provide initial maps of the reaction-diffusion dynamics existing in the rhizosphere, revealing significant differences in the signaling landscape of host and non-host plants before and after wounding, specifically highlighting networks that may inform rhizosphere organization.

## Introduction

The rhizosphere, that narrow zone of soil along plant root surfaces containing bacteria, viruses, fungi, and numerous metabolites, functions as an “external metabolome” (Bais et al., 2006; Badri and Vivanco, 2009; Mendes et al., 2011; Philippot et al., 2013; Huang et al., 2014; Glasser et al., 2017). As with any mutualistic multicellular network, organization is likely critical for metabolome function (Dietrich et al., 2008; Stacy et al., 2014; Stacy et al., 2015; Whiteley et al., 2017). The plant root provides reduced carbon and dioxygen to the rhizosphere (Walker et al., 2003), setting up both ends of life’s universal redox network. Indeed, redox-active phenols, quinones, flavins, and phenazines (Bais et al., 2006; Tomilov et al., 2006; Uteau et al., 2015; Rasmann and Turlings, 2016) are prevalent in the rhizosphere where redox active processes have been highlighted in biofilm biogeography (Stacy et al., 2014), allelopathy (Tomilov et al., 2006), quorum sensing (Fuller et al., 2017), and most notably, in the critical spatiotemporal dynamics of semagenesis ensuring success of the parasitic plants (Keyes et al., 2007; Liang et al., 2016; Fuller et al., 2017). The initial discoveries of the reaction diffusion dynamics of semagenesis (Chang et al., 1986; Fate et al., 1990; Smith et al., 1990) motivated further explorations of model reaction-diffusion systems in the rhizosphere (Taran et al., 2019). Given that the developed models have now greatly expanded how reaction-diffusion networks might contribute to the richly dynamic cellular architecture of the rhizosphere, we sought to develop specific probes to map the processes within and on the surfaces of plant tissues with and without environmental perturbation, specifically focusing on plant wounding.

Virulent strains of *Agrobacterium tumefaciens*, more recently classified as *Rhizobium radiobacter*, genetically transform many dicotyledonous angiosperms and gymnosperms resulting in the formation of tumors at the site of infection (McCullen and Binns, 2006; Nester, 2014; Binns and Zhao, 2020). These soil bacteria transition from a saprophytic state to a pathogenic state in a process that requires (1) identification of and attachment to host cells that are competent to be transformed and (2) the expression of a series of ‘virulence’ (*vir*) genes located on their tumor inducing (Ti) plasmids. The latter process is initiated by multiple small molecule signals, specifically low pH, phenols, and sugars, which appear to be characteristic of host wound environments (McCullen and Binns, 2006; Binns and Zhao, 2020). Simple sugars and phenols from the host then serve as xenognostic signals that initiate activation of the virulence genes through the histidine kinase VirA *via* an integrating AND gate mechanism (Fang et al., 2015).

Fluorescent protein reporters and variants of VirA with altered capacities to recognize the xenognostic signals (McCullen and Binns, 2006; Binns and Zhao, 2020) enable the use of *Agrobacterium* as a dynamic probe of the extracellular milieu surrounding the host tissues. We accordingly have engineered the signal input modules in *Agrobacterium* strains carrying *gfp* under the control of virulence gene promoters to create biosensors. Experiments with these reporter strains provide evidence that *Agrobacterium* cells accumulate around viable host cells at the wound surface. Different tissues of the same plant, including stem, midrib, and leaf, vary in their wound-induced accumulation, consistent with expected phenol content. This wound-induced signaling response is enhanced in mature plants, consistent with the lower susceptibility of seedlings to tumorigenesis (Robbs et al., 1991). Using a VirA mutant strain that responds to sugar independent of phenol (Fang et al., 2015), we show that wound-induced phenol levels accumulate in host tissues and not in non-host controls. In contrast, wound-induced sugar exudation is similar in host and non-host tissues. Taken together, tumor formation appears uniquely dependent on xenognostic phenol release. Indeed, a strain of *Agrobacterium* that is hypersensitive to sugar initiates *vir* gene production, but induces tumors poorly when compared to wildtype *Agrobacterium*, highlighting this critical role of phenols for pathogenesis. These *Agrobacterium* strains then serve as valuable probes of the reaction-diffusion networks at plant wound sites, and most importantly, open strategies for similar constructs in other mutualistic microbes to more broadly define the organization necessary for a functioning rhizosphere.

## Materials and Methods

### Plasmid Construction and *Agrobacterium* Strains

*E. coli* strain XL1-Blue was used for plasmid construction, and a list of all plasmids and strains are found in **Table 1**. pYL355, which contains *virA (Y293F)* and genes flanking both sides of *virA* for specific complementation to allow double crossover, was generated by ligating the *Kpn*I fragment from pQF431 into the *Kpn*I-digested pAW162 (Liu, 2012). The *BamH*I/*EcoR*I fragment was released from pYL355 and ligated with the *BamH*I/*EcoR*I digested pAW190, a derivative of pK18mobsacB, to generate pYL356. *Agrobacterium* strains with *virA(Y293F)* substitution on the Ti plasmid were developed by pK18mobsacB-mediated homologous recombination (Schäfer et al., 1994). To generate YHL300, pYL356 was transformed into 358mxGFP, an engineered A348 strain containing *virE::GFP* as the virulence reporter (Goulian and van der Woude, 2006). The first crossover for *virA(Y293F)* incorporation was selected by kanamycin resistance, and a single colony was chosen and cultivated overnight in LB at 28°C. The saturated culture was 1:200, 1:1,000, and 1:5,000 diluted with fresh LB, and 100 µl of each dilution was spread onto LB plates containing 10% sucrose. Successful incorporation of *virA(Y293F)* was verified by sequencing (Beckman Coulter Genomics), and resulted in YHL300. YHL301 was generated by a single transformation of YHL300 with pMP7605, a plasmid containing *tac*-driven *m-Cherry*. YHL320 was generated similarly with the method described above, but a transformation of pYL356 into AB520, an A348 derivative with a deletion of the sugar transporter MmsB (Zhao and Binns, 2011; Liu, 2012). After the selection on sucrose plates, successful creation of YHL320 was verified by sequencing, and a dual transformation of pMP7605 and pRG182, a plasmid containing VirB-GFP as the virulence reporter, gave rise to YHL324^s+^.

**Table 1:** Bacterial strains and plasmids used in this study.

Strain/plasmid	Relevant characteristics	Reference
*E. coli* strains		
XL1-Blue	*recA1 endA1 gyrA96 thi^-^1 hsdR17 supE44 relA1 lac*[*F’ proAB lacI^q^Z M15* Tn*10* (Tc^r^)]	Stratagene
*A. tumefaciens* (*R. radiobacter*) strains	
A136	Strain C58 cured of Ti plasmid	(Stachel and Nester, 1986; Lee et al., 1998)
A348	A136 containing octopine Ti plasmid pTiA6NC	(Garfinkel et al., 1981)
358mx	A348 with *virE::lacZ*	(Stachel and Nester, 1986; Lee et al., 1992)
358mxGFP	358mx with *lacZ::gfp*, Carb^R^	(Goulian and van der Woude, 2006)
AB650^wt^	358mxGFP containing pMP7605, Carb^R^, Gent^R^	this study
YHL300	358mxGFP, *virA^Y293F^*, Carb^R^	this study
YHL301^ss^	YHL300 containing pMP7605, Carb^R^, Gent^R^	this study
AB520	A348, *ΔmmsB*	(Zhao and Binns, 2011)
YHL320	AB520, *virA^Y293F^*	this study
YHL324^s+^	YHL320 containing pRG182, pMP7605, Kan^R^, Gent^R^	this study
Plasmids		
pKmobsacB	pK19, pSUP102 (RP4 mob) with sacB added	(Schäfer et al., 1994)
pYL355	*virA(Y293F)* in pKmobsacB	this study
pMP7605	Broad-host-range expression vector, IncW, Apr	(Lagendijk et al., 2010)
pRG182	*virB* promoter-*gfp* in pBRR122 (KpnI-XbaI piece from pJET12), Kan^R^	this study

### Culturing, Inoculation, and Co-Cultivation

All *Agrobacterium* inoculums were prepared by growth overnight in LB liquid medium with 100 µg/ml gentamicin for AB650^wt^/YHL301^s+^, or 15 µg/ml kanamycin and 100 µg/ml gentamicin for YHL324^s+^ (Liu, 2012). The overnight bacterial culture is pelleted and resuspended to OD0.2 in half strength *Murashige and Skoog medium* (0.5X MS) buffered to pH5.5 with 50 mM 2-(N-morpholino) ethanesulfonic acid (MES) and mixed with 0.005% triton, and then incubated with plant material for 10 min (mature Nt or Zm), 5 min (young Nt seedlings), or 1 min (Sa seedlings) for inoculation. The bacterial suspension was washed off and the plant material was placed on three types of co-cultivation medium in petri dishes: glucose plates, AS plates and glycerol plates, which contain 0.5X MS medium (pH5.5) supplemented with 56 mM glucose, 100 µM AS, or 100 mM glycerol, respectively. Glucose plates are used for measuring AS equivalents released from plant material, AS plates are for measuring glucose equivalents, and glycerol plates are for measuring a combination of the signals released from the plant material (induction potential). The AS dose responses and glucose dose responses were performed in triplicate in 0.5X MS (pH5.5) liquid medium supplemented with either 56 mM glucose or 100 mM glycerol for measuring AS dose response, or supplemented with 100 µM AS or no AS for measuring the glucose dose response. The range of concentration titrated was 0, 0.1, 1, 10, 100, 300 µM AS or 0, 0.01, 0.1, 1, 10, 100 mM glucose. In all treatments, 15 µg/ml or 50 µg/ml of kanamycin was added to liquid culture or agar plates of YHL324 ^s+^ incubation to maintain the GFP containing plasmid pRG182. Plasmid maintenance was secure in culture, but less stable in planta.

### Preparation of Plant Material and Growth Media

Seeds of tobacco (*Nicotiana tabacum* cv. Havana 38) and *Zea mays* were purchased from LEHLE Seeds. Prior to germination, seeds were surface sterilized with 50% bleach and 0.1% triton for 10 min, and then washed with excess sterile water for 4–6 times. Seedlings were grown on 0.5X MS + 1% sucrose solid medium (containing filtered sucrose to avoid glucose contamination through autoclaving) until two true leaves emerged and expanded. Some seedlings (>3) were inoculated for examination and the others (>3) were transplanted to soil for obtaining mature plants. Seeds of *Striga asiatica* were received from USDA under quarantine, surface sterilized with 3% chromic acid, 50% bleach and 0.1% triton for 10 min, and 70% ethanol for 2 min, being rinsed thoroughly with sterile water between each treatment. The seeds were then incubated in sterile water at 28°C for 7–10 days before germination was induced hydroponically in 0.1 mM KCl solution with 10 nM Strigol. Haustorium development was induced hydroponically in 0.1 mM KCl solution with 10 µM DMBQ (Chang and Lynn, 1986).

Wounding mature tobacco or two-month-old maize plants was done by cutting stem/midrib explants with razor blades, or leaf explants with a cork borer (**Figure S1**). Wounded seedlings of tobacco or *S. asiatica* were created by pinching hypocotyl/root with pointed tweezers, or cutting the cotyledon with small scissors. Wounding of *Kalanchoe diagremontiana* was performed with a wooden dowel scraping the cuticle of the leaf 3–4 times.

All plant-*Agrobacterium* co-cultivation took place on 0.5X MS plates at pH5.5. The pH is buffered to pH5.5 because we found that without buffer the plant tissues changed the pH of the growth media and caused sporadic and/or large fluctuations in the virulence response (data not shown).

### Flow Cytometry

A population of *Agrobacteria* were washed off the plant tissue by vortexing the co-cultivated plant tissue in 0.5X MS liquid medium (pH5.5) with confocal confirmation of effective bacterial removal. The GFP fluorescence of each bacterium was measured by flow cytometry and a population of 10,000 bacteria was counted from each sample with each condition being performed in triplicate. The average GFP fluorescence per bacterium was plotted. GFP fluorescence was excited by a blue sapphire laser at 488 nm laser with emission BP at 525/50 using the long pass 505 nm filter. m-Cherry fluorescence was excited by a green laser at 532 nm with the emission BP at 610/20 using the long pass 600 nm filter.

### Fluorescence Imaging

A Zeiss Laser Confocal Scanning Microscope LSM 510 META was used for fluorescence imaging. Argon/2 laser at 488 nm was used to excite GFP and a filter of 505–530 nm was applied for detecting GFP emission. Helium Neon later at 543 nm was used to excite m-Cherry and a filter of 585–615 nm was applied to detect m-Cherry emission. DIC was applied to acquire bright field images. All representative images are presented as a merge of GFP, m-Cherry, and DIC channels.

## Results

### Construction of *Agrobacterium* as a Biosensor

We constructed three *Agrobacterium tumefaciens* strains as biosensors to evaluate the spatiotemporal distribution of phenols and sugars at plant wound sites (**Table 2**). We began by obtaining a previous strain, 358mxGFP (Goulian and van der Woude, 2006), that contains the gene for green fluorescent protein (GFP) as a replacement for the virulence gene *virE2* in strain A348^wt^. This strain functions as a nonvirulent fluorescent reporter of plant wound signal reception (Liu, 2012). Additionally, the gene encoding mCherry fluorescence protein under the constitutive Tac promoter located in a separate plasmid, pMP7605 (Lagendijk et al., 2010), was added to this strain to give AB650^wt^. The discovery of a signal integration node in VirA (Fang et al., 2015) led to a simple mutation that separated the co-dependence on sugar and phenol for induction of virulence signals. Insertion of VirA^Y293F^ for wild-type VirA in AB650^wt^ generated sugar-sensitive strain YHL301^ss^, which can be induced by sugar or phenol alone. A third strain, YHL324^s+^, was prepared by inserting VirA^Y293F^ in place of wild-type VirA in AB520. AB520 is a strain carrying a deletion of the multiple monosaccharide transporter B (*ΔmmsB*) (Zhao and Binns, 2011), which enhances the sensitivity of *Agrobacterium* to *vir*-inducing sugars by increasing the relative sugar concentration in the periplasm (Hu et al., 2012). A separate plasmid, pRG182, carrying VirB-GFP was added into YHL324^s+^ to enable sugar sensing independent of phenols, but with significantly higher sensitivity than YHL301^ss^. Although YHL324^s+^ has a different GFP source than AB650^wt^ and YHL301^ss^ and has therefore been left out of any quantitative comparisons between AB650^wt^ and YHL301^ss^, it has proved useful as a tool for reporting bacterial position due to its signal hypersensitivity. Taken together, strains AB650^wt^, YHL301^ss^, and YHL324^s+^ provide the resources for evaluating the relative levels of sugars and phenols at the wound site of hosts and non-host plants.

**Table 2:** Characteristics of *A. tumefaciens* biosensor strains used in this study.

Strain	Genotype	GFP promoter	mCherry promoter	Virulence	Sugar sensitivity	Phenol sensitivity
AB650^wt^	VirA^wt^	*virE*	*tac*	–	+	+
YHL301^ss^	VirA^Y293F^	*virE*	*tac*	–	++	++
YHL324^s+^	VirA^Y293F^ *ΔmmsB*	*virB*	*tac*	+	+++	++

**Figure 1:**
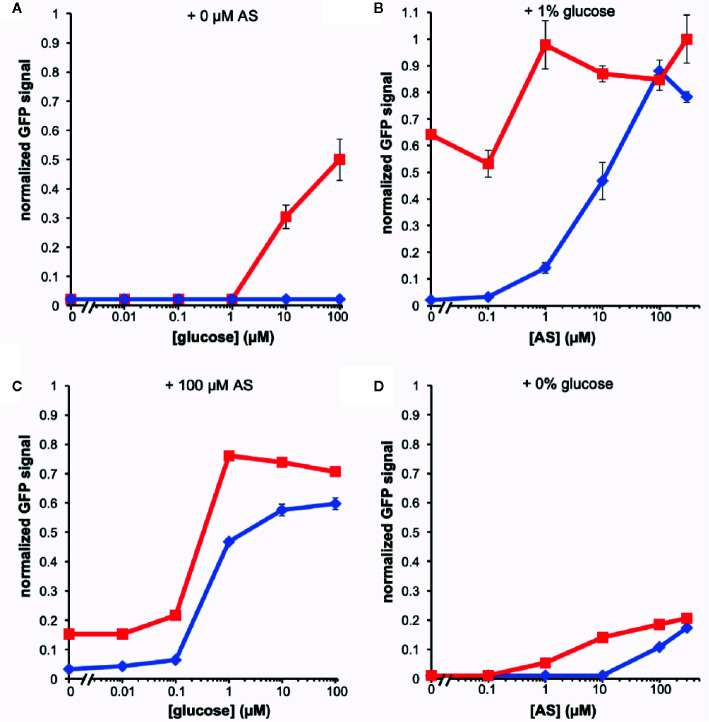
Dose responses of *A. tumefaciens* fluorescent strains to acetosyringone (AS) or glucose. Upon addition of AS or glucose, *virE::gfp* [AB650^wt^ (blue diamonds), YHL301^ss^ (red squares)] signal was measured and normalized to the maximal value of YHL301^ss^ GFP signal. Glucose dose responses with no AS **(A)** or 100 µM AS **(C)** in the growth medium and AS dose responses with 1% glucose **(B)** or 1% glycerol **(D)** in the growth medium. Glycerol is used as a non-inducing carbon source in the absence of glucose. Each data point represents the average of three biological replicates and error bars show ± SE.

The fluorescence of the AB650^wt^ and YHL301^ss^ strains in response to acetosyringone (AS) and glucose was measured independently [100 µM AS or 56 mM (1%) glucose] in liquid media (**Figure 1**). While we obtained similar data with the YHL324^s+^ strain (**Figure S1**), we recognize that this is an imperfect comparison due to the different GFP sources and have therefore focused on the AB650^wt^ and YHL301^ss^ strains. A summary of the sensitivity of these two strains is reported as the ED_50_ in **Table 3**. While AB650^wt^ requires AS independent of the concentration of glucose (**Figure 1A**), YHL301^ss^ is strongly induced by glucose alone (**Figure 1B**) and displays enhanced sensitivity to phenols (**Figure 1C**). Maximal induction and sensitivity to AS in both strains are enhanced in the presence of glucose (**Figure 1B vs D**).

**Table 3:** ED_50_ of *A. tumefaciens* biosensors to acetosyringone (AS) or glucose.

Strain	ED_50_ to AS/µM		ED_50_ to glucose/mM	
	1% glucose	no glucose	100 µM AS	no AS
AB650^wt^	8	100	0.6	–
YHL301^ss^	0.2	3–4	0.2	10

### Agrobacterium Cells Accumulate in the Apoplast of *N. tabacum* at Wound Sites

**Figure 2:**
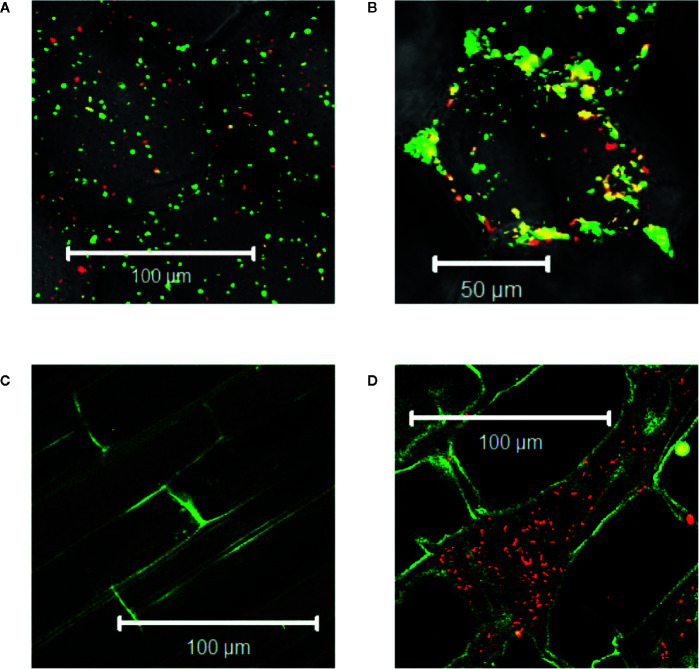
*Agrobacterium* cells colonizing plants localize to the apoplast. YHL324^s+^ colonizing the pith tissue of tobacco stem explants on the surface **(A)** or 60 µm below the surface **(B)**. Unfortunately, co-expression of both markers was not uniform in the absence of selection, limiting quantitative assessment. Nevertheless, unwounded **(C)** and wounded **(D)** tobacco seedlings co-cultivated with AB650^wt^ reveal distinct bacterial colonization in the wounded plant. The cells walls of the tobacco seedlings auto-fluoresce, allowing for clear delineation of plant cell structure.

*Agrobacterium* will naturally colonize plants and form benign bio-films on root surfaces (Heindl et al., 2014) and it is generally accepted that antimicrobial substances released at the wound can limit colonization (Akinsulire et al., 2008; Taye et al., 2011; Meij et al., 2018). We show here that *Agrobacterium* cells are able to colonize wounded mature tobacco explants (**Figure 2A**). Unfortunately, without selection either or both markers can be lost in a portion of our bacteria and we cannot use quantitative expression under these conditions; nevertheless, co-incubation with our YHL324^s+^ strain on 0.5X MS plates confirms the presence of bacteria in optical sections 60-80 µm below the apical surface, corresponding to several cell layers into the wound (**Figure 2B**). Wildtype *Agrobacterium* cells (AB650^wt^) are not detected in unwounded two-week-old tobacco seedlings (**Figure 2C**), but appear in the apoplast of wounded tobacco seedlings (**Figure 2D**). In general, young tobacco seedlings do not induce GFP production similarly to mature tobacco plants, which we explore further. These analyses suggest that a wounding event either lowers a physical barrier and/or produces chemical attractants such as sugars or phenols for *Agrobacterium* colonization of viable cells (Loake et al., 1988).

### Virulence-Inducing Phenols Accumulate at Living Cells in a Wound

**Figure 3:**
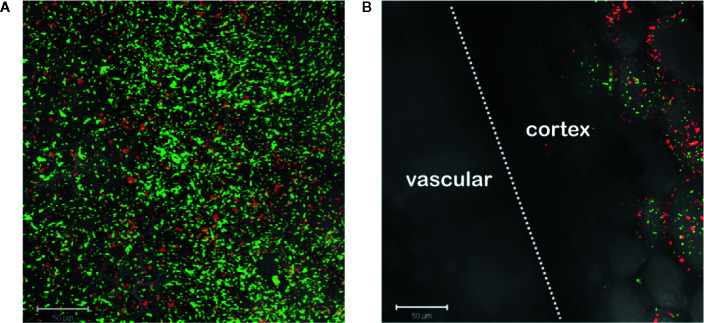
*Agrobacterium* colonize into the apoplast of tobacco explants within two days of co-cultivation. YHL324^s+^ colonize on the surfaces **(A)** and 50 µm below the surface **(B)** of the stem explants. The white dashed line indicates the junction between vascular tissue and the cortex tissue.

Agrobacterium chemotaxis has been recognized and suggested to be required for functional colonization and infection (Chesnokova et al., 1997; Merritt et al., 2007). Micromolar concentrations of sugars and nanomolar amount of wound phenolics have been reported to attract *Agrobacterium* to the plant wounds (Ashby et al., 1988; Loake et al., 1988; Winans, 1992). As phenolic monomers and acidic pH are characteristic of plant cell walls and vacuoles, it is possible that phenols and sugars can be released from breached cells, however, *Agrobacterium* needs to target living cells for transformation. We compared *Agrobacterium* localization between vascular tissue (comprised of a majority of dead cells) and the adjacent pith or cortex tissue (comprised of living cells) at a cut site in the tobacco stem. Using YHL324^s+^, we observed colonization and induction on the surface of the stem explants (**Figure 3A**), however the bacteria were not detected in the vascular channels even though these wide conduits are readily accessible to the bacteria. Instead, accumulation occurred 50–60 μm below the surface in the adjacent cortex tissue within two days of co-cultivation (**Figure 3B**, **Figure S3**). While this was a reproducible result, there remains the possibility that these observations are due to alternative differences between these cell types, including the transmittance efficiency, and this will need to be addressed with further experimentation. Population in the apoplast of the living tissue in the cortex is necessary for successful transformation, but how that behavior may be mediated by xenognostic phenol/sugar signals or other molecules impacting *Agrobacterium* localization will now need to be determined.

### Agrobacterium Behaviors at Monocot and Dicot Wound Sites Differ

**Figure 4:**
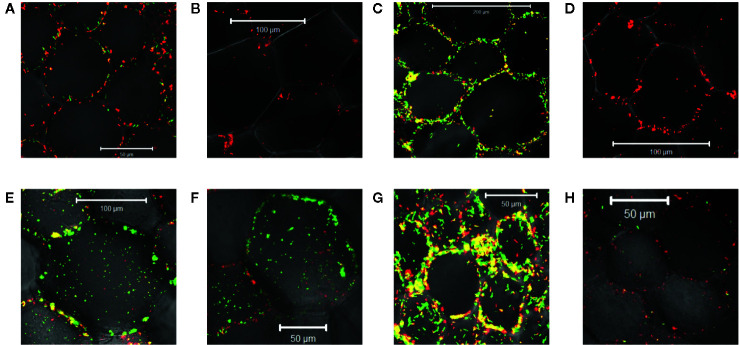
Virulence induction events in tobacco vs. maize midrib explants. AB650^wt^ with explants of tobacco **(A)** and maize **(B)** on glycerol plates; YHL301^ss^ with explants of tobacco **(C)** and maize **(D)** on glycerol plates; YHL324^s+^ with explants of tobacco **(E)** and maize **(F)** on glycerol plates; AB650^wt^ with tobacco explants on glucose plates **(G)**, and AB650^wt^ with tobacco explants on AS plates **(H)**.

Monocots initially appeared resistant to *Agrobacterium* pathogenesis and indeed the plants lack sufficient amounts of one or more *vir* inducers upon wounding (Hooykaas, 1989; Smith and Hood, 1995). The successful transformation of rice enhanced with exogenous AS supported that assertion (Hiei et al., 1994; Vijayachandra et al., 1995; Nishimura, 2020). Wall phenol structures from monocots differ widely from those of dicots and even among monocot species (Usami et al., 1988; Messens et al., 1990). Additionally, monocot plant growth regulators or secondary metabolites have been reported to inhibit the process of *vir* gene induction (Sahi et al., 1990; Zhang et al., 2000; Maresh et al., 2006). Given these results, we were interested in the behavior of our three engineered strains of *Agrobacterium* at monocot wound sites. Using tobacco as a dicot control and maize as a monocot non-host, midrib explants were inoculated with each of these bacterial strains. AB650^wt^ was induced by the dicot (**Figure 4A**) but not the monocot (**Figure 4B**); YHL301^ss^ was strongly induced by the dicot (**Figure 4C**), but not the monocot (**Figure 4D**); and YHL324^s+^ was induced by both (**Figures 4E, F**), suggesting that both tobacco and maize wound sites contain sufficient sugar to induce the sugar responsive YHL324^s+^ strain. It is important to note that the YHL324^s+^ strain does retain virulence while the AB650^wt^ and YHL301^ss^ strains do not (see **Table 2**), which could contribute to differential induction seen in these experiments.

To test whether sugar might limit induction in the tobacco wound site, we co-cultivated the mid-rib explants with AB650^wt^ on 0.5X MS medium supplemented with 56 mM glucose, a saturating sugar concentration for virulence induction. These conditions strongly induced AB650^wt^ (**Figure 4G**), but when supplemented with 100 μM AS and no sugar, AB650^wt^ was only minimally induced (**Figure 4H**). These results suggest that these wounded tobacco midrib explants fail to induce high virulence expression (as in **Figure 4A**) due to low levels of the xenognostic sugar in the wound, as opposed to low phenol levels, and motivated our efforts to quantify GFP expression in each *Agrobacterium* strain *via* flow cytometry.

### Cell Counts of Agrobacterium at Wound Sites

**Figure 5:**
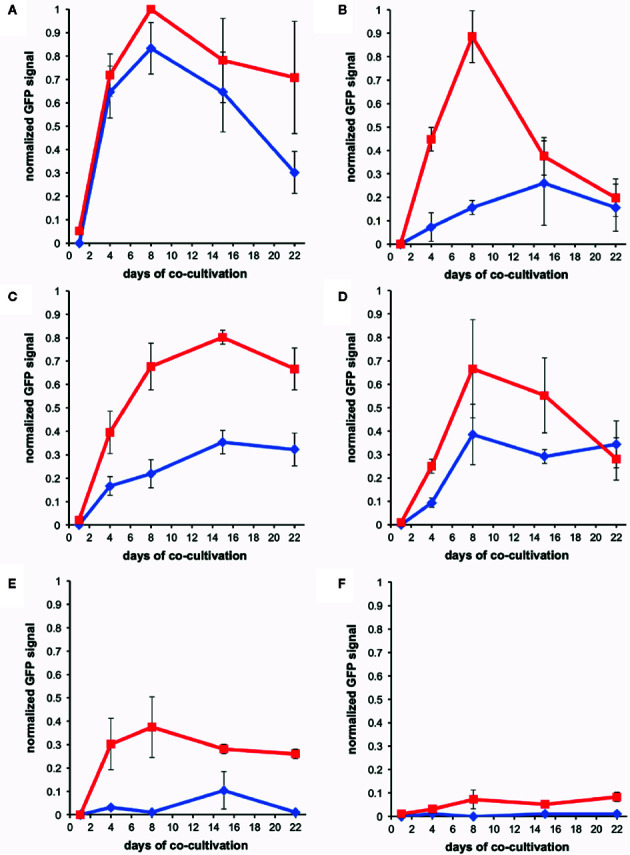
Quantification of wound-induced virulence inducers by co-cultivation. Bacteria were co-incubated with plant explants, vortexed, and GFP intensity was determined using flow cytometry (see Methods). Experiments were performed in triplicate and error bars represent ± SE. AB650^wt^ (blue diamonds) or YHL301^ss^ (red squares) with tobacco midrib explants on glucose plates **(A)**, maize midrib explants on glucose plates **(B)**, tobacco midrib explants on acetosyringone (AS) plates **(C)**, maize midrib explants on AS plates **(D)**, tobacco midrib explants on glycerol plates **(E)**, and maize midrib explants on glycerol plates **(F)**.

Bacterial cells harvested from co-cultivation with plant tissues were analyzed *via* flow cytometry. While absolute quantification is limited by washing efficiency, we sought a relative comparison between similar bacterial strains. Our engineered strains vary in sensitivity to phenol and sugar concentrations as defined in **Figure 1** (Liu, 2012). As in **Figure 1**, we did not include YHL324^s+^ because the production of GFP in these cells was from a different source than AB650^wt^ and YHL301^ss^. We compared host vs nonhost plants conditioned by wounding, organ/tissue type, and age (**Table 4**) with the AS and glucose equivalents estimated according to the ratio of YHL301^ss^ induction to AB650^wt^ induction. To determine whether phenols are limiting for *vir-*induction in a particular plant tissue, co-cultivations with different bacterial strains were performed in the presence of saturating glucose concentrations. Similarly, to determine whether *vir*-inducing sugars are limiting in plant tissues, co-cultivations were conducted in the presence of saturating levels of the inducing phenol, AS. The complete comparisons are available in supplemental information (**Figures S4–6**), including how YHL324^s+^ responds in this assay, and a typical example in each test group contained in **Figure 5** is discussed in the sections below.

**Table 4:** Summary of the quantified phenolic, sugar, or combined effect of both inducers, released from tobacco and maize as conditioned by wounding, organ/tissue types, or age.

Plant Condition	*vir*-inducers		6-month-old tobacco			2-month-old maize		2-week-old (young) tobacco		
			Stem	Midrib	Leaf	Midrib	Leaf	Stem	Midrib	Leaf
**Wounded**	**Phenol**	**Conc.**	10-100 µM	10-100 µM	5-10 µM	1-5 µM	5-10 µM	0-0.1 µM	0-0.1 µM	0-0.1 µM
		**Peak length**	>18 days	>18 days	>18 days	<11 days	<7 days	<7 days	<7 days	<7 days
	**Sugar**	**Conc.**	0.5-1 mM	0.5-1 mM	0.1-0.5 mM	0.1-0.5 mM	0.5-1 mM	0-0.1 mM	0-0.1 mM	0.1-1 mM
		**Peak length**	<18 days	<18 days	<18 days	<18 days	<18 days	>7 days	>7 days	>7 days
	**Induction potential**		0.1	0.03	0.08	0.01	0.01	0.01	0.02	0.01
**Unwounded**	**Phenolic**		ND	ND	ND	ND	ND	ND	ND	ND
	**Sugar**		ND	ND	ND	ND	ND	ND	ND	ND

The concentration and duration of phenol or sugar exudation is quantified according to GFP response (**Figures S4–6**) with **Figure 1** as the reference. The induction potential is presented as how AB650^wt^ with wt signal sensitivity responded to plant tissue without exogenous inducers (**Figures S4–6**), normalized to the maximum induction level seen from YHL301^ss^ across all the treatment groups. The vir-inducers exuded by unwounded plant tissue tobacco seedlings were not detected (ND) by AB650^wt^ or YHL301^ss^.

### Xenognostic Phenols in Wounded Hosts vs. Nonhosts

The wound-released active phenols were compared according to the virulence expression on glucose plates where glucose concentrations are saturating. For these experiments, explants were obtained from tobacco leaves on the midrib near the petiole and always incubated in the same orientation on plates. Similarly, maize explants were excised from the midrib about halfway between the plant stem and leaf tip. On the tobacco midrib explants (**Figures 5A, B**), YHL301^ss^ and AB650^wt^ responded at similar rates and to similar levels, indicating the explants released saturating concentrations of xenognostic phenols (**Figure 1A**). The maize midrib explants (**Figure 5B**) induced YHL301^ss^ four times higher (0.8 vs. 0.2) and twice as fast as AB650^wt^ (8 days vs. 15 days), confirming that the maize explants release lower concentrations of active phenols. The observation that the peak of induction on maize explants disappeared within 4-7 days is consistent with previous reports that, in contrast to dicots, monocots have weaker short-lived wounding responses (Savatin et al., 2014; Hayta et al., 2019). Initial data with AB650^wt^ cultures pre-induced with 100 µM AS on glycerol 0.5X MS plates suggest that it takes up to 8 days after removal of AS for the pre-existing GFP to be completely turned over (**Figure S8**). Therefore, the duration of phenols released was estimated by measuring for how many days the YHL301^ss^ induction was above the threshold of 0.2 (**Table 4**).

### Xenognostic Sugars in Wounded Hosts vs. Nonhosts

On plates containing 100 µM AS, the ratio of responses between the two *Agrobacterium* strains is very similar between wounded tobacco and maize explants (**Figures 5C, D**), suggesting that tobacco and maize release similar amount of sugars at wound sites. This result suggests that the different susceptibility to *Agrobacterium* infection between host dicots and non-host monocots rests with wound-induced phenols rather than sugars. YHL324^s+^ however responded very differently on these AS plates relative to the glucose plates, with induction higher than YHL301^ss^ in all cases (**Figures S4–S6**). Given that it only takes 10–20 mM glucose for complete induction of YHL324^s+^ (see **Figure S2**), the AS plates may allow YHL324^s+^ to perform normally because of the enhanced glucose sensitivity. Finally, testing each of these strains on glycerol plates confirmed both the designation of the YHL301^ss^ strain as sugar sensitive and the observation that dicots can induce a virulence response at conditions where monocots cannot (**Figures 5E, F**).

### Induction by Young *Nicotiana tabacum* Seedlings Mirrors That of the Parasitic Plant Striga Asiatica

**Figure 6:**
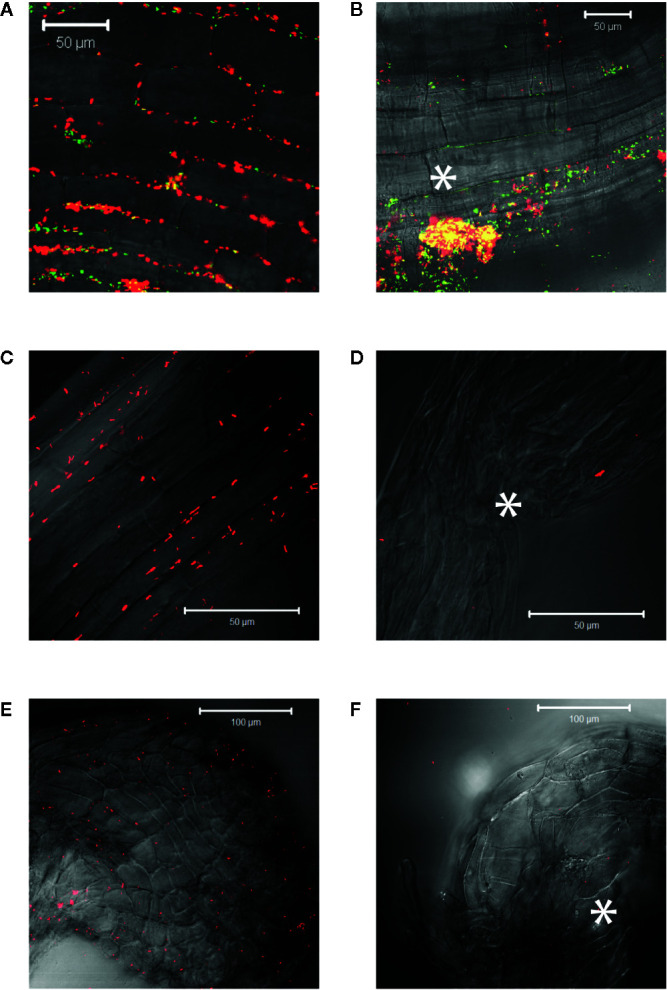
Virulence induction events reveal similarities between young seedlings of different species. Strain YHL324^s+^ was co-cultivated with two-week old tobacco seedlings on glycerol plates and an unwounded root **(A)** and a wounded root **(B)** are shown. A similar experiment was performed with five-day-old *S. asiatica* seedlings and YHL324^s+^ on glycerol plates and representative images are shown: unwounded root of a five-day-old *S. asiatica* seedling **(C)**, a wounded root of a *S. asiatica* seedling **(D)**, a haustorium of a *S. asiatica* seedling **(E)**, and a wounded haustorium of a *S. asiatica* seedling **(F)**. White asterisks indicate approximate wound locations.

The process of signal generation, or semagenesis, in parasitic plants depends on low cell wall phenol content in the parasite. Here the production of H_2_O_2_ at the parasite root tip is proposed to create a mild wound response *via* peroxidase oxidation of wall phenols in the host in order to generate the haustorial inducing benzoquinone at the host/parasite interface (Chang and Lynn, 1986; Keyes et al., 2007). The parasite has been shown to have low phenol content in its own cell wall to avoid self-response, and this concept has been generalized to root meristematic tissue of other dicots including tobacco (Fuller et al., 2017). In contrast to mature tobacco tissue, two-week-old tobacco seedlings failed to induce AB650^wt^ or YHL301^ss^ (data not shown). These young plants weakly induced YHL324^s+^ when unwounded (**Figure 6A**) but do show greater induction upon wounding (**Figure 6B**) (Liu, 2012). Consistent with the hypothesis that the parasite appears to have weaker phenol content, seedlings of the dicot *Striga* *asiatica* do not induce expression in any of the three strains when unwounded (**Figure 6C**), wounded (**Figure 6D**), following haustorium induction (**Figure 6E**), or even following wounding of the haustorium (**Figure 6F**) on glycerol plates. These results are consistent with a critical role for wall phenols in the host age dependence in *Agrobacterium* pathogenesis.

### Hypersenstive Agrobacterium Strains Differentially Infect Dicots

**Figure 7:**
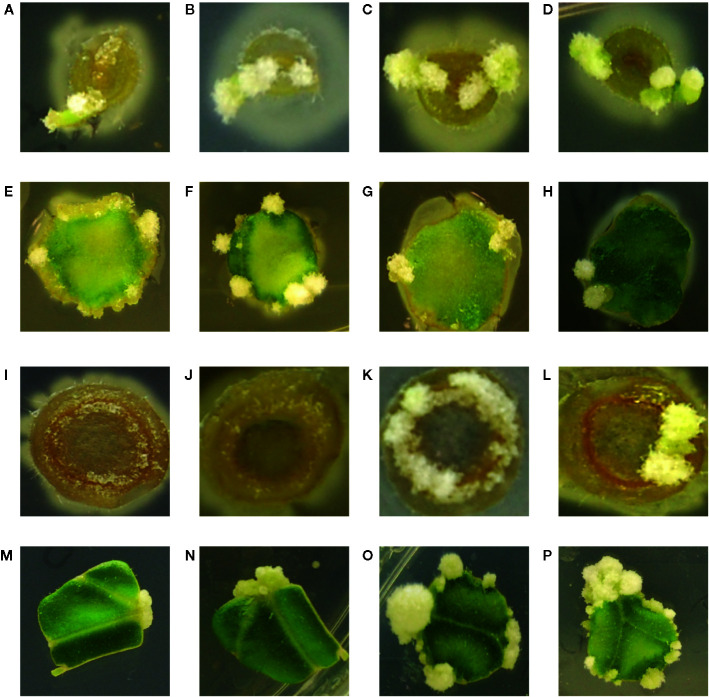
Crown gall tumor induction on tobacco explants. Using mature plants started out in tobacco midrib explants were co-cultivated with YHL320^s+^ on glucose plates **(A)**, and glycerol plates **(B)**, or with A348^wt^ on glucose plates **(C)** and glycerol plates **(D)**; tobacco leaf explants with YHL320 ^s+^ on glucose plates **(E)** and glycerol plates **(F)**, or with A348^wt^ on glucose plates **(G)** and glycerol plates **(H)**; tobacco stem explants with YHL320 ^s+^ on glucose plates **(I)** and glycerol plates **(J)**, or with A348^wt^ on glucose plates **(K)** and glycerol plates **(L)** for 51 days on 0.5X MS plates. Additionally, tobacco leaf explants are shown co-cultivated with A348^wt^ for 20 days **(M)**, 34 days **(N)**, or 78 days **(O, P)**. Representative photos are shown and quantification of tumor induction rate is found in **Table 5**.

While the hypersensitive strain YHL324^s+^ is strongly induced by maize explants, published data indicate that while T-DNA transfer can occur in maize leaf tissues, tumorigenesis was not observed (Shen et al., 1993). These authors suggest this result could arise from the absence of cell division in maize leaf tissues even after transformation. After two weeks of co-cultivation on tobacco explants, however, YHL320^s+^ (YHL324^s+^ without the GFP containing plasmid) or wild type strain A348^wt^ initiated tumor growths on the leaves and midribs of tobacco (**Figures 7A–H**) (Liu, 2012). Interestingly, YHL320^s+^ caused the stem explants to display necrosis, including a reduction in pith tissue and, possibly for that reason, no tumor production (**Figures 7I, J**). In contrast, A348^wt^ bacteria did not cause such severe necrosis and these stem segments were able to form tumors (**Figures 7K, L**). Including glucose in the media aided tumor formation with A348^wt^ in tobacco, but not for YHL320^s+^ (**Table 4**).

**Table 5:** Tumor induction rate on tobacco explants co-cultivated with A348^wt^ and hypersensitive YHL320^s+^.

Strains	Plates	Stem	Midrib	Leaf
A348^wt^	Glucose	96% (22/23)	72% (20/32)	85% (22/26)
	Glycerol	38% (9/24)	44% (14/32)	40% (10/25)
YHL320^s+^	Glucose	No data	20% (7/35)	64% (14/22)
	Glycerol	No data	64% (21/33)	92% (23/25)

**Figure 8:**
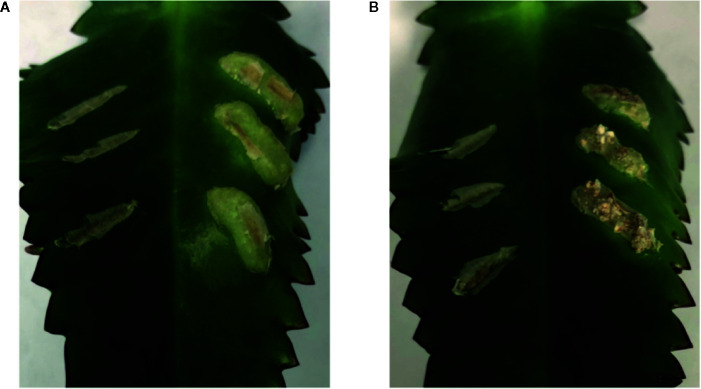
Both VirA^wt^ and VirA^Y293F^ *Agrobacterium* induce crown gall tumors. *Kalanchoe diagremontiana* leaves were inoculated with A348^wt^ **(A)** and YHL320^s+^ **(B)** in triplicate on the right of each leaf, while the wounds to the left were left as controls. Tumors for A348^wt^ appeared larger and more uniform.

To further evaluate the relationship between increased sugar sensitivity and successful tumorigenesis, we inoculated *Kalanchoe diagremontiana* with A348^wt^ and YHL320^s+^ strains (**Figure 8**). While the wild type *A. tumefaciens* produced typical tumors (**Figure 8A**), the tumors produced by strains containing the VirA^Y293F^ mutation appeared small and irregular (**Figure 8B**), indicating a further phenotypic difference between the two strains. The preliminary location of the tumors is also informative. We observed that the tumors in tobacco formed preferentially at cambium cells in the junction between two tissue types (**Figures 7I–L**), possibly due to the presence of stem cells (**Figures 7C–H**). These tumors appeared to arise in a polar fashion (**Figures 7M, N**) and only when the co-cultivation lasted for more than 35 days did the opposite cut end and additional spots around the cut edge of the leaf disks initiate tumor growth (**Figures 7O, P**). While this localization pattern supports the hypothesis that it is potentially the rapidly dividing cells generated at a wound site that are susceptible to *Agrobacterium*-mediated transformation, further experimentation probing the precise locations of the cell divisions will be necessary.

## Discussion

Mapping the behavior of *Agrobacterium* within hosts tissues has the potential to correlate models for plant wounding (Savatin et al., 2014) with the evolution of plant pathogenesis (Lin et al., 2014; Hayta et al., 2019). The xenognosin receptor of *A. tumefaciens* functions as an AND gate, requiring both simple sugars and phenols to initiate pathogenesis (Fang et al., 2015). We show that while the exudation of sugars is similar between certain tissues of host (tobacco leaves) and non-host (maize leaves) plants, wound-localized xenognostic phenols in maize are orders of magnitude lower and occur as a transient spike rather than the sustained exudation observed in tobacco. *In situ* imaging identifies the accumulation of *A. tumefaciens* cells around viable host tissues. Accumulation is localized below the wound surface, rather than the adjacent vascular cells at the same depth, and is consistent with the pathogen targeting viable cells (Dubravina et al., 2005). This contrasting behavior highlights the differing wounding profiles between host and non-host and the well-known weak wound responses (Hiei et al., 1994) and low levels of *vir*-inducing exudates from monocots (Hooykaas, 1989).

**Figure 9:**
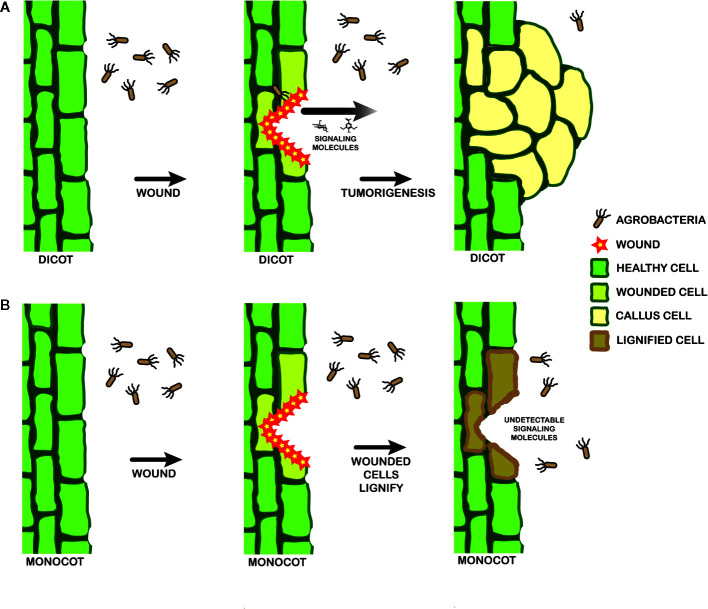
Model for signal landscape of monocots and dicots. In dicots **(A)**, wounding events release detectable signals into the rhizosphere, which can be recognized by a pathogenic *Agrobacterium*. Chemotaxis follows, leading to DNA transfer and tumorigenesis. Wounding in monocots **(B)**, in contrast, leads to lignification in the cell walls and a lack of strong signals by a pathogenic *Agrobacterium*.

Several strains of *A. tumefaciens* were constructed to further understand the molecular origins of these pathogen behaviors. YHL301^ss^ contains a mutation within the xenognostic VirA receptor, allowing it to function as an OR gate where either sugar or phenol alone induce virulence gene expression (Fang et al., 2015). YHL324^s+^ contains the same mutation as well as the deletion of the multiple monosaccharide transporter B (*δmmsB*), a deletion which confers hypersensitivity (Zhao and Binns, 2011). Virulence gene expression is induced in both of these strains by dicot explants, and YHL324^s+^ is also induced by monocot tissues. However, this hypersensitive strain did not induce tumors in wounded maize plants, suggesting that pathogen incompatibility in monocots may be limited by other intrinsic factors than those responsible for the initial stages of virulence gene induction (Maresh et al., 2006; Maresh et al., 2007). As outlined in **Figure 9**, wound healing in dicots involves dedifferentiation and proliferation to repair the wound, but monocot cells lose the ability to dedifferentiate very early in development (Graves et al., 1988). Instead, monocots form a lignified ring of hardened cells that quickly seals the wound from invading microbes (Sood et al., 2011; Miedes et al., 2014; Lee et al., 2019), a process that likely limits plant transformation(Graves et al., 1988). Indeed, *Agrobacterium*-mediated transformation of monocot cells has been more successful in embryonic calli and immature embryos (Chan et al., 1993; Ishida et al., 1996; Cheng et al., 1997), while wounding in dicots appears to generate significant numbers of competent cells at the wound site (Binns, 1990; Ikeuchi et al., 2013).

Spatiotemporal mapping with *Agrobacterium* then opens a window into the complex and dynamic behaviors of the communities that naturally inhabit the plant wound site. More generally, the bacterial strains reported here may be extended to probe the reaction-diffusion networks that contribute to specifying the complex cellular order of the rhizosphere (Taran et al., 2019). Such methods for mapping the dynamic behaviors of the epiphytes, symbionts, pathogens, and other integral members of this complex multicellular community will become increasingly important for achieving a more sustainable agriculture and addressing the food challenges we will face in our changing global climate.

## Data Availability Statement

The raw data supporting the conclusions of this article will be made available by the authors, without undue reservation.

## Author Contributions

SL and BP initiated this project in the lab of DL, and BP developed new aspects of this research in his own lab. Y-HL significantly contributed to the creation of the constructs. BP, SL, NW, AM, and JA performed the experiments reported. BP and DL wrote the manuscript with contributions from SL, AM, and AB.

## Funding

This work was funded by the University of Richmond School of Arts & Sciences which provided startup funds for BP in addition to his National Institute of Health K12 GM000680 Fellowship in Research and Science Teaching (FIRST) Institutional Research and Academic Career Development Award (IRACDA). The undergraduate students AM, NW, and JA were all funded through the University of Richmond research grants. The work of DL, SL, and Y-HL were funded through The National Science Foundation award IOS # 1423862.

## Conflict of Interest

The authors declare that the research was conducted in the absence of any commercial or financial relationships that could be construed as a potential conflict of interest.
