# Supplementary material for: Mapping Reaction-Diffusion Networks at the Plant Wound Site With Pathogens
Source: Front Plant Sci. 2020 Jul 16;11:1074. doi: 10.3389/fpls.2020.01074 (PMC7379035; doi:10.3389/fpls.2020.01074)
Supplement: Supplementary file 1 [file DataSheet_1.pdf]

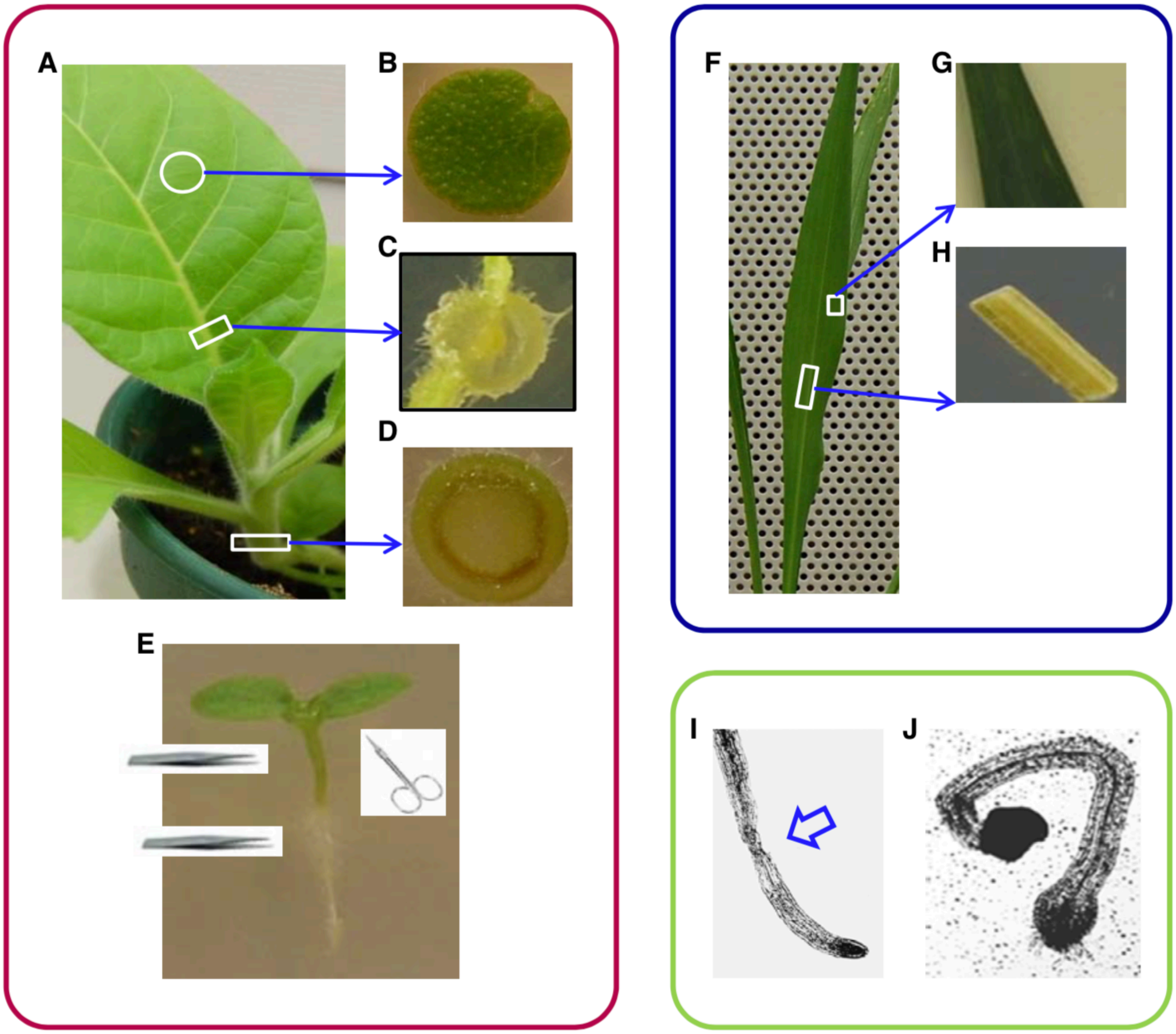

Figure S1. *Illustration on how plant tissues were prepared for co-cultivation.* Flowered tobacco plants (A) were cut into leaf explants (B) by cork borer, mid-vein explants (C) and stem explants (D) by razor blade. 1-2-weeks-old tobacco seedlings were wounded by cutting the cotyledons by small scissors, or pitching the hypocotyls and root by pointed tweezers (E). 2-months-old maize plants (F) were cut into leaf explants (G) and mid-vein explants by razor blades. Maize stem explants were not prepared because at this stage of the plant, the upright stalk was mainly composed of leaves wrapped in a concentric manner. Therefore, the stalk slices would be a mixture of mid-vein and leaf tissue. 1-day-old *S. asiatica* radicals were conditioned by pitching (I) or haustorium induction (J) before exposure to *A. tumefaciens*.

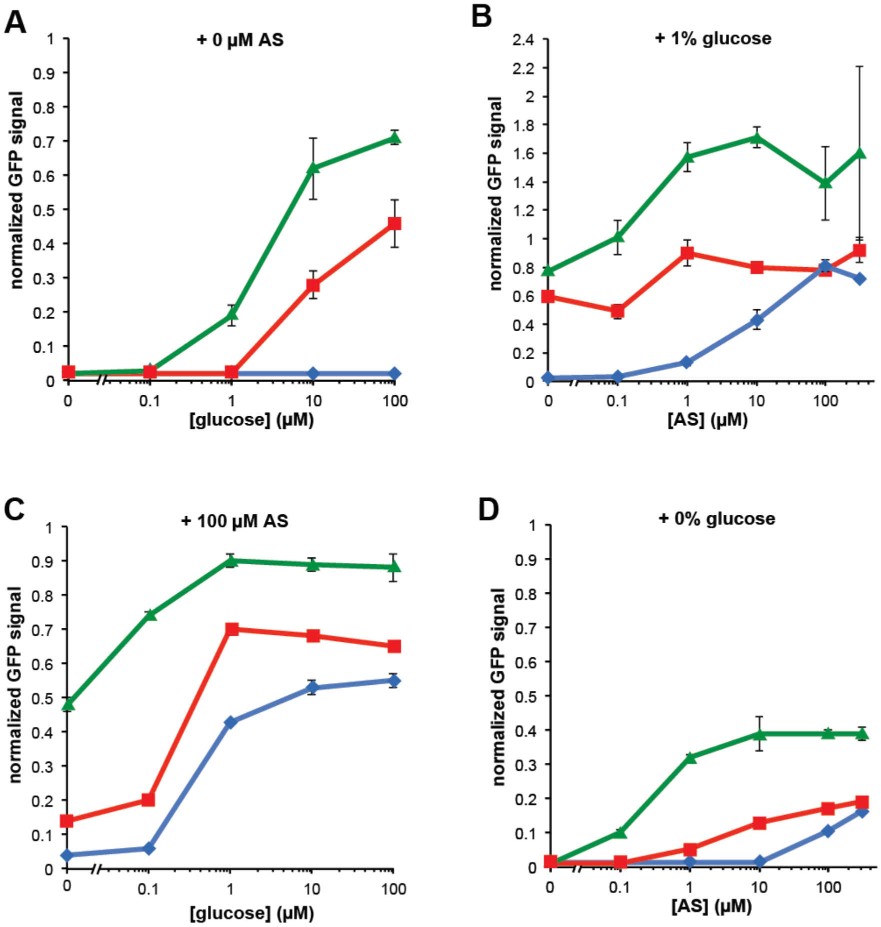

Figure S2. *Complete dose responses of the three A. tumefaciens strains to AS or glucose.* Upon addition of AS or glucose, virE::gfp [AB650^{wt} (blue diamonds), YHL301^{ss} (red circles)] or VirB-GFP [YHL324^{s+} (green triangles)] signal was measured and normalized to the maximal value of YHL301^{ss} GFP signal. Glucose dose responses with 100 μM AS (A) or no AS (C) in the growth medium and AS dose responses with 1% glucose (A) or 1% glycerol (B) in the growth medium. Glycerol is used as a non-inducing carbon source in the absence of glucose.

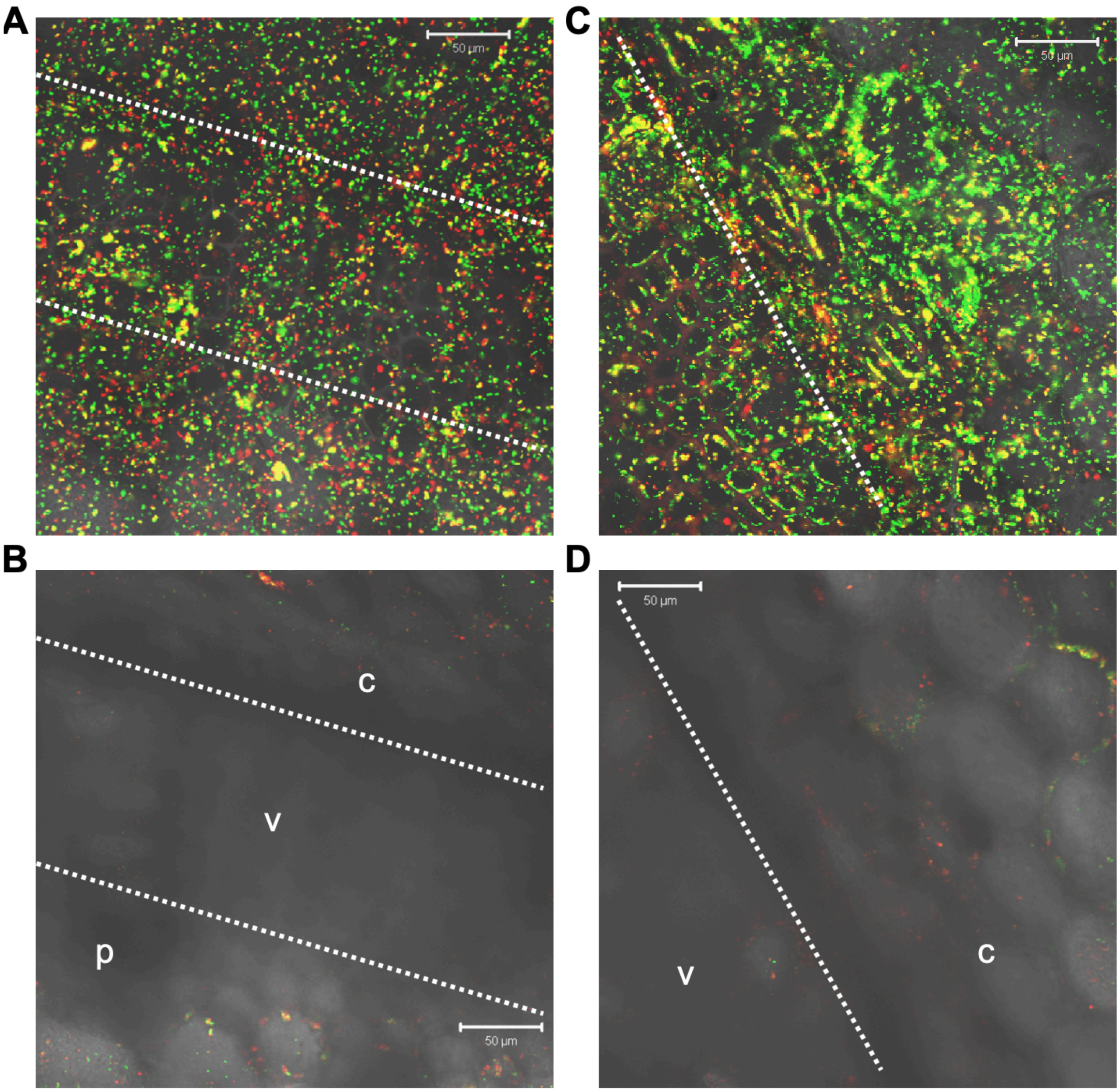

Figure S3. Agrobacterium *colonize into the apoplast of tobacco explants within two days of co-cultivation.* YHL324^{s+} colonize on the surfaces (A, C) and 50 µm below the surface (B, D) of stem explants. The white dashed line separates the junction between vascular tissue (v), the cortex tissue (c), and the pith (p).

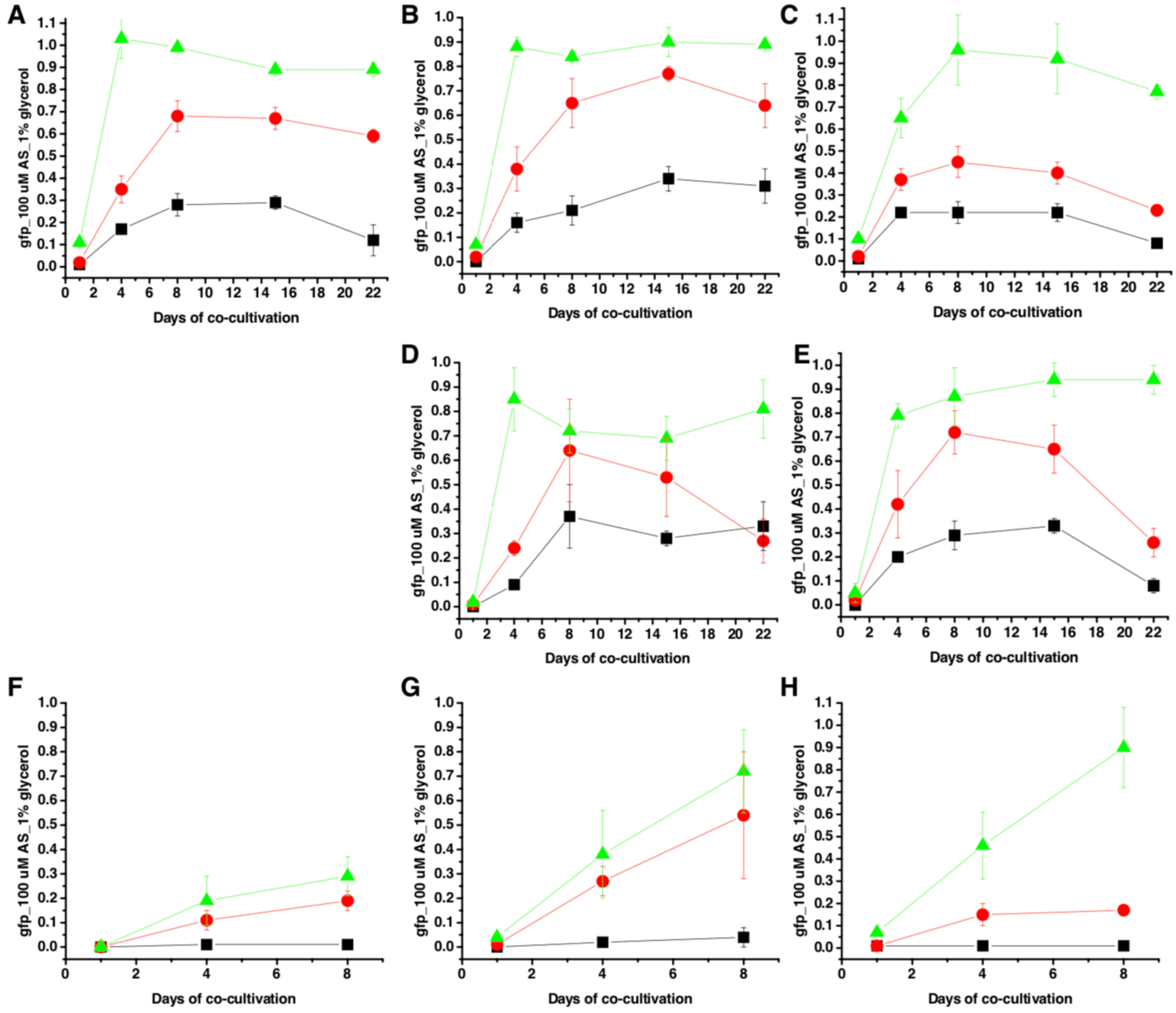

Figure S4. *Quantification of wound-induced sugars by co-cultivation on AS plates.* AB650 (black squares), YHL301 (red circles), and YHL324 (green triangles) co-cultivated with tobacco stem explants (A), tobacco mid-vein explants (B) and tobacco leaf explants (C), maize mid-vein explants (D), and maize leaf explants (E), hypocotyls of wounded tobacco seedlings (F), leaves of wounded tobacco seedlings (G) and roots of wounded tobacco seedlings (H).

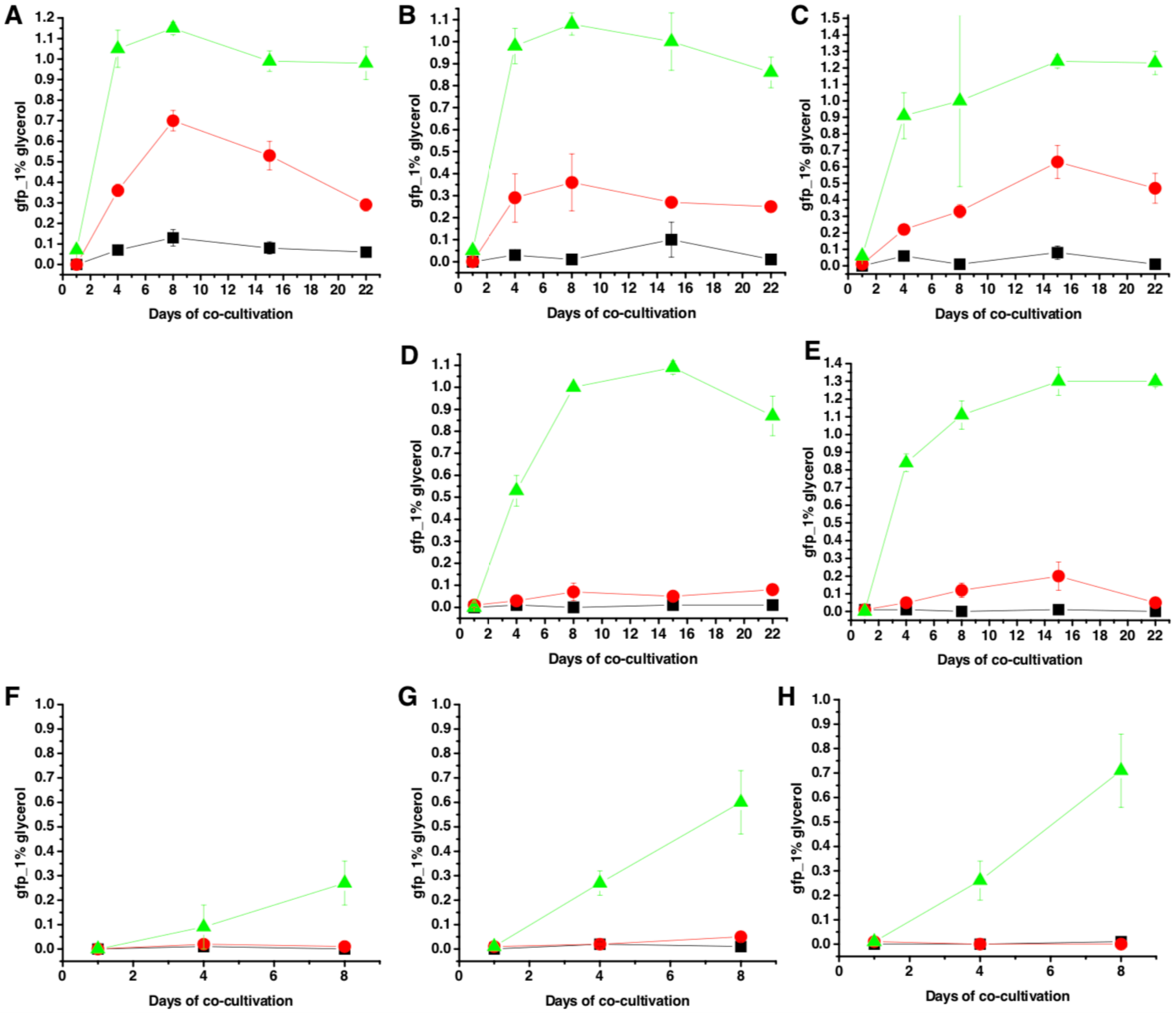

Figure S5. *Quantification of the combinatorial effects of both phenols and sugars by co-cultivation on glycerol plates.* AB650 (black squares), YHL301 (red circles), and YHL324 (green triangles) co-cultivated with tobacco stem explants (A), tobacco mid-vein explants (B) and tobacco leaf explants (C), maize mid-vein explants (D), maize leaf explants (E), hypocotyls of wounded tobacco seedlings (F), leaves of wounded tobacco seedlings (G) and roots of wounded tobacco seedlings (H).

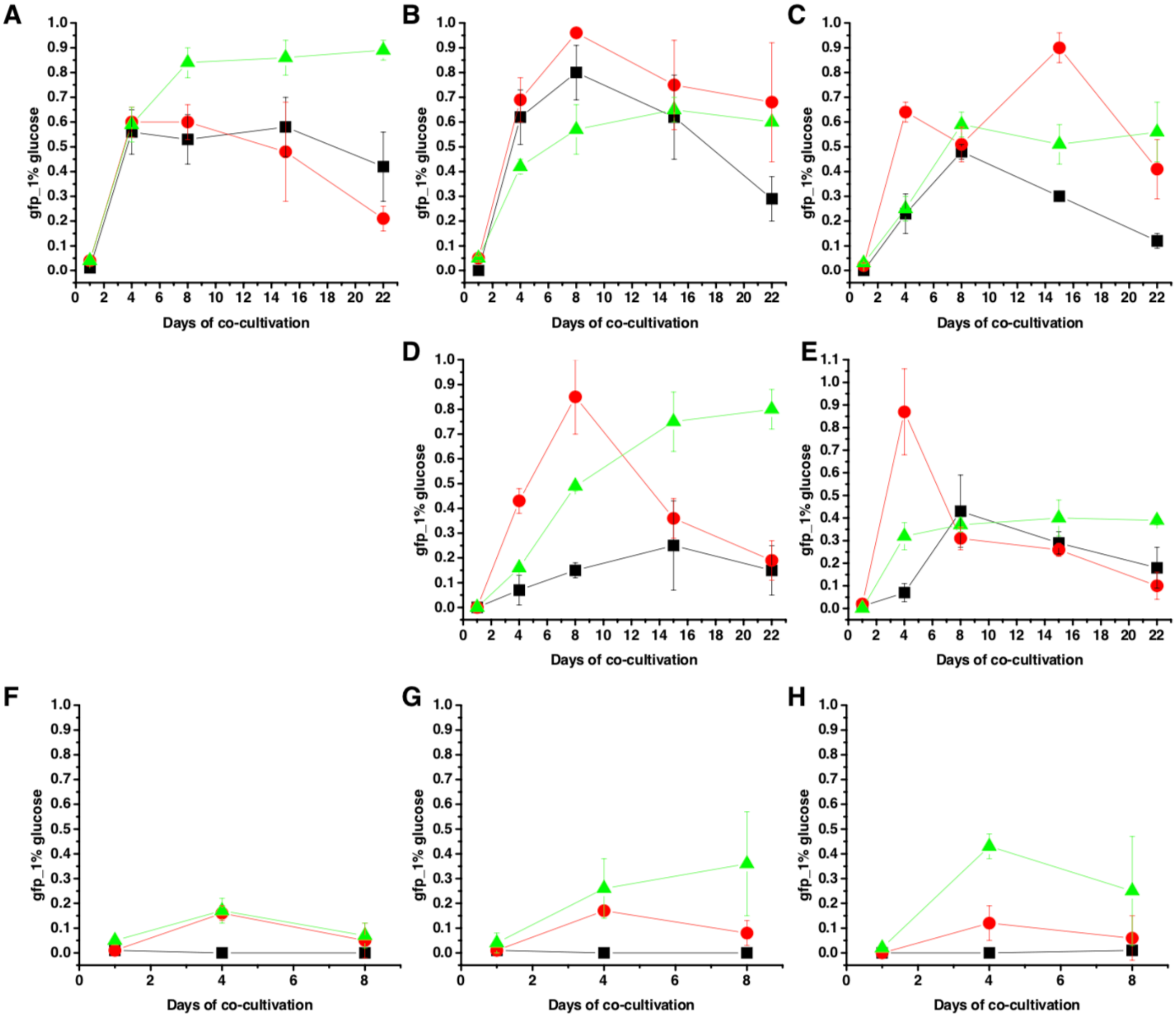

Figure S6. *Quantification of the combinatorial effects of both phenols and sugars by co-cultivation on glucose plates.* AB650 (black squares), YHL301 (red circles), and YHL324 (green triangles) co-cultivated with tobacco stem explants (A), tobacco mid-vein explants (B) and tobacco leaf explants (C), maize mid-vein explants (D), maize leaf explants (E), hypocotyls of wounded tobacco seedlings (F), leaves of wounded tobacco seedlings (G) and roots of wounded tobacco seedlings (H).

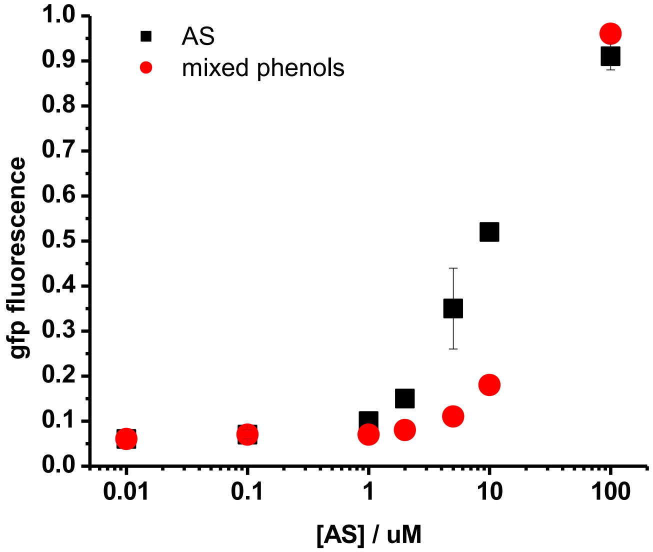

Figure S7. *Determination of whether phenols bind to VirA in a cooperative mode.* The dose response of AB650 to a single inducer, AS, (black squares), or to a mixture of eight vir-inducing phenolic compounds (red circles) with each one at a concentration of an eighth of the corresponding AS concentration.

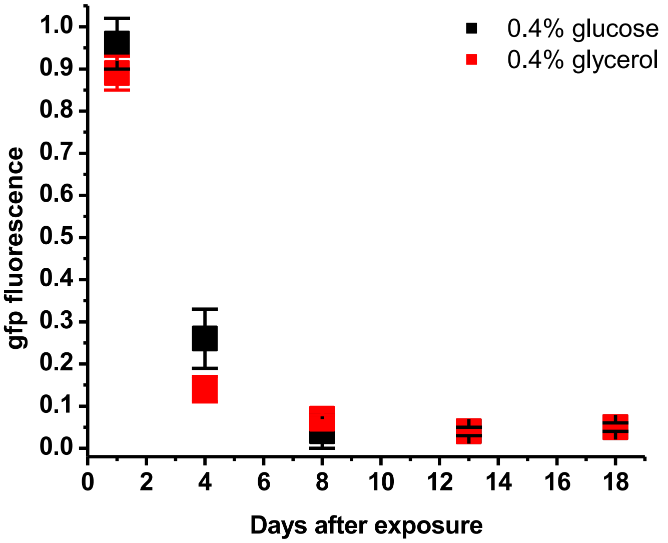

Figure S8. *Evaluation of how rapidly gfp proteins are disposed in A. tumefaciens.* AB650 was grown in ½ MS + 0.4% glucose medium under pre-inducing condition with 100 µM AS from day 0 to day 1. At day 1, AS was removed by pelleting the bacteria, wash the bacteria with ½ MS two times and then resuspend the bacteria in ½ MS + 0.4% glucose (black squares), or ½ MS + 0.4% glycerol (red squares). The fluorescence level of remaining gfp was monitored at indicated days.
